# MSF-UBRW: An Improved Unbalanced Bi-Random Walk Method to Infer Human lncRNA-Disease Associations

**DOI:** 10.3390/genes13112032

**Published:** 2022-11-04

**Authors:** Lingyun Dai, Rong Zhu, Jinxing Liu, Feng Li, Juan Wang, Junliang Shang

**Affiliations:** School of Computer Science, Qufu Normal University, Rizhao 276826, China

**Keywords:** lncRNA-disease associations, linear neighborhood similarity, Gaussian interaction profile, logistic function, unbalanced bi-random walk

## Abstract

Long-non-coding RNA (lncRNA) is a transcription product that exerts its biological functions through a variety of mechanisms. The occurrence and development of a series of human diseases are closely related to abnormal expression levels of lncRNAs. Scientists have developed many computational models to identify the lncRNA-disease associations (LDAs). However, many potential LDAs are still unknown. In this paper, a novel method, namely MSF-UBRW (multiple similarities fusion based on unbalanced bi-random walk), is designed to explore new LDAs. First, two similarities (functional similarity and Gaussian Interaction Profile kernel similarity) of lncRNAs are calculated and fused linearly, also for disease data. Then, the known association matrix is preprocessed. Next, the linear neighbor similarities of lncRNAs and diseases are calculated, respectively. After that, the potential associations are predicted based on unbalanced bi-random walk. The fusion of multiple similarities improves the prediction performance of MSF-UBRW to a large extent. Finally, the prediction ability of the MSF-UBRW algorithm is measured by two statistical methods, leave-one-out cross-validation (LOOCV) and 5-fold cross-validation (5-fold CV). The AUCs of 0.9391 in LOOCV and 0.9183 (±0.0054) in 5-fold CV confirmed the reliable prediction ability of the MSF-UBRW method. Case studies of three common diseases also show that the MSF-UBRW method can infer new LDAs effectively.

## 1. Introduction

Long-non-coding RNAs (lncRNAs) are long chains composed of nucleotides, with a wide range of actions and complex mechanisms. They get involved in many critical regulatory processes [1,2,3,4] and have attracted the attention of many life scientists and biologists in recent years. Studies have found that mutations and disorders of lncRNAs are bound up with the occurrence of human diseases [5,6], including AIDS [7], diabetes [8], Alzheimer’s disease [9], and many types of cancer, such as breast cancer [10], prostate [11], hepatocellular [12], and bladder cancer [13]. Many associations between lncRNAs and diseases and how they interact have also become a good breakthrough for researchers to understand the pathogenesis of diseases from the molecular level.

Although the research on identifying human lncRNA-disease associations (LDAs) progresses rapidly, the precise principles behind it remain largely unclear, such as transcriptional regulation, multi-biological processes, and molecular mechanisms of various diseases [14]. Predicting the undiscovered LDAs can help people figure out the pivotal factor of lncRNAs in biological processes, thus helping with the diagnosis, treatment, and prognosis of diseases. Using computational models to predict potential LDAs takes far less time and cost than biological experiments. Therefore, it is of great significance to study computational models to reveal new LDAs for further experimental verification. Scientists have done a lot to the research of lncRNA-disease relationship, and many excellent predictive models have appeared [15,16,17]. Existing models for predicting LDAs mainly fall into two categories: machine learning-based methods and biological network-based methods [18]. Machine learning-based methods play an important role in predicting LDAs. Classifiers can be trained based on the characteristics of known disease-associated lncRNAs and those of unknown disease-associated lncRNAs. Candidate lncRNAs can be ranked in line with the differences of biological characteristics. Lan et al. [19] developed a supervised method: LDAP, which integrated multivariate biological data. In this method, the bagging support vector machine (SVM) was trained to predict LDAs. Multiple training datasets are constructed by bagging method, and each dataset is trained by SVM to generate multiple weak classifiers, which vote on the category of test samples. Chen et al. [20] proposed a computational method: Laplacian Regularized Least Squares for LDA (LRLSLDA). This method was based on a semi-supervised learning framework to predict new LDAs and achieved reliable performance. However, LRLSLDA still has some limitations. For example, there are many parameters in the method, and it is very difficult to determine the optimal parameters. In addition, for the same LDA pair, two different scores can be obtained from the lncRNA space and the disease space, respectively. How to efficiently combine the two scores has become a current research topic. Gao et al. designed a method: Multi-Label Fusion Collaborative matrix factorization (MLFCMF) [21] to identify LDAs. First, the inner links between lncRNAs and diseases were improved and the hidden information was discovered by multi-label learning. Second, the fusion method was used to learn the multi-label information. Finally, potential LDAs were inferred by collaborative matrix factorization. Fu et al. [17] reconstructed the LDA matrix by the optimized low-rank matrices to identify latent LDAs. Lu et al. [22] proposed a method to recover informative features by principle components analysis and complement the LDA matrix derived from the inductive matrix completion. For the machine learning-based methods, the main challenge is how to select useful biometrics to train the classifier. Therefore, integrating multiple data resources can effectively improve prediction performance. Biswas et al. [23] designed a novel method for predicting potential LDAs based on matrix factorization. The model integrated known LDAs, experimentally verified gene-disease associations, gene-gene interaction data, and the profiles of lncRNAs and genes. The bi-clustering method was used to identify lncRNA modules and non-negative matrix factorization (NMF) was used to reveal potential LDAs.

In recent years, the outstanding performance of network-based methods in predicting LDAs has aroused the researchers’ interest. Many excellent algorithms have emerged based on the hypothesis that functionally similar lncRNAs may be related to diseases with similar phenotypes. For example, Sun et al. [24] proposed a computing method, namely RWRlncD. In this study, after the establishment of the LDA network, the disease similarity network (DSN) and the lncRNA similarity network (LSN), RWRlncD predicted the potential LDAs by randomly walking on the LSN. It is worth noting that RWRlncD is robust to different parameters. As more LDAs and more accurate measures of the lncRNA functional similarity become available, the prediction ability of RWRlncD will be improved. Zhou et al. [25] also designed a novel model to identify potential LDAs. This model integrated three networks (i.e., the miRNA-associated lncRNA-lncRNA crosstalk network, the DSN and the known LDA network) into one network and conducted random walks on it. However, the method is only applicable to lncRNAs with known lncRNA–miRNA interactions. In addition, the incomplete coverage of the lncRNAs crosstalk network and the LDA network may reduce the prediction performance of the model. Xie et al. [26] developed a method to infer new LDAs. First, the features of lncRNAs and diseases were mapped to the features of local-constraint by location-constrained linear coding, and then the initial correlation matrix and the acquired features of lncRNAs and diseases were mixed up by the label propagation strategy. Xie et al. [18] also used the weighted K-nearest known neighbors algorithm (WKNKN) method to solve the problem with rare known LDAs and applied the linear neighbor similarity (LNS) to reconstruct the DSN and LSN. In 2020, Ref. [27] designed a method to reveal potential LDAs. The method combined the heat spread algorithm and probability diffusion algorithm to reallocate resources, and used unbalanced bi-random walks to infer new LDAs.

However, these methods have some drawbacks. For example, most methods only introduce Gaussian Interaction Profile (GIP) kernel similarity, which makes the prior information used for prediction too simple and single. In response to this question, we propose a new method called MSF-UBRW to infer potential LDAs based on multiple similarities fusion and unbalanced bi-random walk. First, the lncRNA functional similarity matrix is obtained from known LDA matrix. Second, the GIP kernel similarity of lncRNAs is calculated derived from known LDAs, and the logistic function is used to adjust the similarity of the lncRNA network. The same is true for the disease network. Third, linear fusion is performed for the above two similarities of lncRNAs and diseases, respectively. Then, the initial association probability matrix is calculated by WKNKN. Next, the pairwise linear neighborhood similarities of lncRNAs and diseases are calculated. Finally, LDAs are inferred by bi-randomly walking with different steps on the lncRNA network and the disease network. The main highlights of the MSF-UBRW method are as follows:

(1) Linear fusion was performed for lncRNA functional similarity and GIP kernel similarity of lncRNAs, as well as for disease semantic similarity and GIP kernel similarity of diseases. In addition to that, logistic functions are constructed from known LDAs to improve the topology structure of networks.

(2) So far, very few LDAs have been identified, which results in a sparse LDA matrix. WKNKN is used to preprocess the known LDA matrix to solve the sparse problem and obtain the association probability matrix.

(3) The linear neighbor similarity is applied to reconstruct the DSN and LSN.

The MSF-UBRW method achieves the reliable AUC values with 0.9391 and 0.9183 (±0.0054) based on leave-one-out cross validation (LOOCV) and 5-fold cross validation (5-fold CV), respectively. In addition, case studies of three common diseases (prostate cancer, esophageal squamous cell carcinoma (ESCC), and small cell lung cancer (NSCLC)) further prove the prediction ability of the MSF-UBRW method. Experimental results demonstrate that MSF-UBRW is an effective and reliable method for identifying potential LDAs.

## 2. Materials and Methods

### 2.1. Datasets

The known LDA dataset is downloaded from the public database LncRNADisease [28]. Due to the database upgrade, you can also download the new dataset from the LncRNADisease V2.0 database. We can provide the data set used in the experiment, if you need. After removing the non-human items and duplicated data, we finally get the known human LDAs, including 115 kinds of lncRNAs and 178 kinds of diseases. Then, $L=\left\{l_{1},l_{2},\cdots ,l_{n_{l}}\right\}$ denotes the lncRNA set, and $D=\left\{d_{1},d_{2},\cdots ,d_{n_{d}}\right\}$ is the disease set. We can describe the known LDAs by constructing a 115×178 dimensional adjacency matrix $Y\in R^{n_{l}\times n_{d}}$. If the lncRNA $l_{i}$ is related to the disease $d_{j}$, $Y_{i,j}=1$; otherwise, $Y_{i,j}=0$.

### 2.2. Disease Similarity

The disease similarity is usually described by directed acyclic graphs (DAGs) in recent research [18,21,27,28]. In this study, the disease similarity is obtained by the following steps. First, the MeSH descriptor for each disease is downloaded from the U.S. National Library of Medicine. Second, based on the precise classification and semantic information provided by the MeSH descriptor, we use the Directed Acyclic graphs (DAGs) to calculate the disease semantic similarity. Let $DAG\left(D_{i}\right)=D\left(D_{i},N\left(D_{i}\right),E\left(D_{i}\right)\right)$ is the DAG of the disease $D_{i}$. In the expression above, the node set $N(D_{i})$ contains all the nodes, and the edge set $E(D_{i})$ contains all the direct links between nodes in the $DAG(D_{i})$. For each disease $D_{i}$, the semantic value can be defined as follows:

(1) $$D_{sum}\left(D_{i}\right)=\sum_{d\in DAG(D_{i})}D_{D_{i}}\left(d\right),\;$$

(2) $$D_{D_{i}}\left(d\right)=\left\{\begin{array}{cc} 1 & if\;d=D_{i},\\ max\left\{\delta \times D_{D_{i}}\left(d^{{}^{\prime }}\right)|d^{{}^{\prime }}\in children\;of\;d\right\} & if\;d\neq D_{i}. \end{array}\right.\;$$

**Figure 1:**
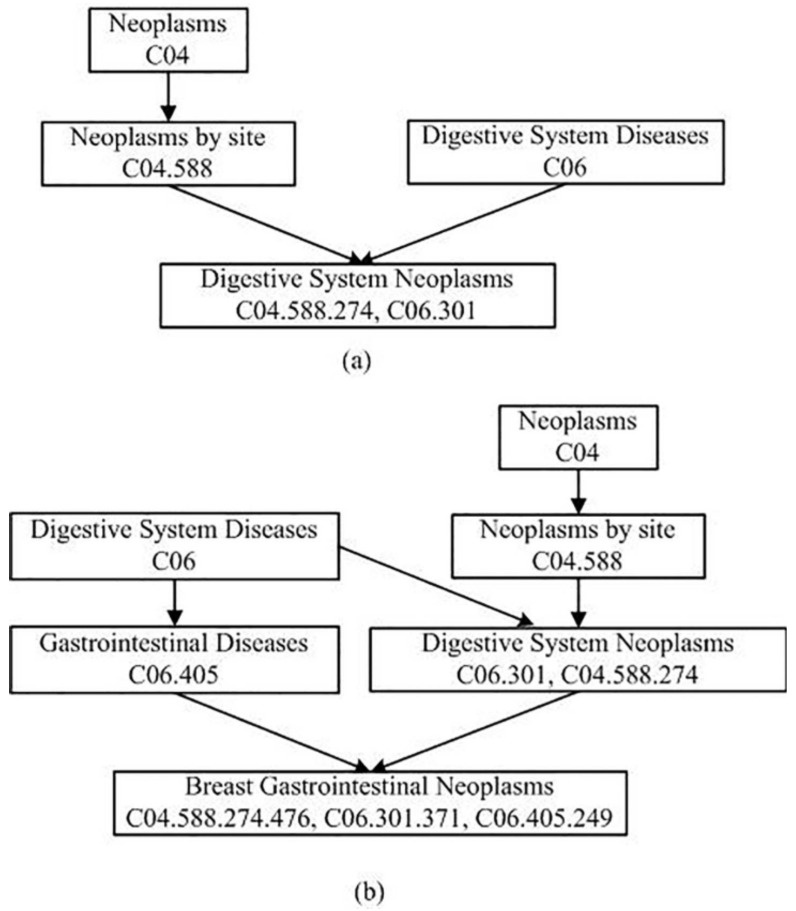
DAGs of digestive system neoplasms and breast gastrointestinal neoplasms. (**a**) digestive system neoplasms. (**b**) breast gastrointestinal neoplasms.

δ∈[0,1] in (2) denotes the semantic contribution factor. According to the current research methods, we set δ to be 0.5. The node’s contribution to itself is defined as 1.0. The DAGs of the Digestive System Neoplasms and the Breast Gastrointestinal Neoplasms are illustrated in Figure 1. According to Figure 1, the semantic values of these two diseases can be calculated using Formulas (1) and (2). For Digestive System Neoplasms, $D_{sum}\left(D_{i}\right)=1.0$ (Digestive System Neoplasms)+0.5 (Digestive System Diseases) + 0.5 (Neoplasms by Site) + 0.5×0.5 (Neoplasms)=2.25. For Breast Gastrointestinal Neoplasms, $D_{sum}\left(D_{i}\right)=1.0$ (Breast Gastrointestinal Neoplasms) + 0.5 (Gastrointestinal Diseases) + 0.5×0.5 (Digestive System Diseases) + 0.5 (Digestive System Neoplasms) + 0.5×0.5 (Neoplasms by Site) + 0.5×0.5×0.5 (Neoplasms) = 2.625.

Previous studies have shown that the more similar the structures of two diseases’ DAGs are, the greater the semantic contribution value will be. The semantic similarity between two diseases $d_{i}$ and $d_{j}$ can be calculated as the following formula:

(3) $$S_{dis}\left(d_{i},d_{j}\right)=\frac{\sum_{t_{i}\in \left(DAG\left(d_{i}\right)⋂DAG\left(d_{j}\right)\right)}\left(D_{d_{i}}\left(t_{i}\right)+D_{d_{j}}\left(t_{i}\right)\right)}{D_{SUM}\left(d_{i}\right)+D_{SUM}\left(d_{j}\right)},\;$$

where $S_{dis}$ is the disease semantic similarity matrix.

As shown in Figure 1, there are four kinds of nodes in the gather $DAG\left(d_{i}\right)⋂DAG\left(d_{j}\right)$. They are Neoplasms, Neoplasms by Site, Digestive System Diseases, and Digestive System Neoplasms. Therefore, $\sum_{t_{i}\in \left(DAG\left(d_{i}\right)⋂DAG\left(d_{j}\right)\right)}\left(D_{d_{i}}\left(t_{i}\right)\right)$ = 1.0 (Digestive System Neoplasms)+0.5 (Digestive System Diseases)+0.5 (Neoplasms by Site) +0.5×0.5 (Neoplasms) = 2.25, $\sum_{t_{i}\in \left(DAG\left(d_{i}\right)⋂DAG\left(d_{j}\right)\right)}\left(D_{d_{j}}\left(t_{i}\right)\right)$ = 0.5×0.5 (Digestive System Diseases) +0.5 (Digestive System Neoplasms)+0.5×0.5 (Neoplasms by Site)+0.5×0.5×0.5 (Neoplasms) = 1.125. Finally, the semantic similarity between Digestive System Neoplasms and Breast Gastrointestinal Neoplasms is calculated according to the Formula (3): $S_{dis}\left(d_{i},d_{j}\right)\;=$$\frac{2.25\;+\;1.125}{2.25\;+\;2.625}\;=\;0.6923$.

### 2.3. LncRNA Similarity

In previous studies, Chen et al. [29] proposed and tested the assumption that functionally similar lncRNAs are usually related to diseases with similar phenotypes, and vice versa. In 2015, Chen et al. [29] obtained the functional similarity between two lncRNAs by calculating the similarity between two sets of diseases associated with these two lncRNAs. For example, $l_{1}$ and $l_{2}$ are two different lncRNAs. It is assumed that $l_{1}$ and $l_{2}$ are associated with two sets of diseases ${\mathrm{Dis}}_{1}=\left\{d_{1},d_{2},\cdots ,d_{m}\right\}$ and ${\mathrm{Dis}}_{2}=\left\{d_{1},d_{2},\cdots ,d_{n}\right\}$, respectively. The similarity between a disease *d* (d∈Dis) and its set including *k* diseases can be defined as:

(4) $$S_{dis}\left(d,Dis\right)=max\left(S_{dis}\left(d,d_{i}\right)\right),\;$$

where $d_{i}\in Dis,1⩽i⩽k$. The similarity between $l_{1}$ and $l_{2}$ can be defined as the sum of similarities between all diseases of the sets with the respective other set, normalized by the size of the sets:

(5) $$S_{l}\left(l_{1},l_{2}\right)=\frac{\sum_{i=1}^{m}S_{dis}\left(d_{1i},Dis_{2}\right)+\sum_{j=1}^{n}S_{dis}\left(d_{2j},Dis_{1}\right)}{m+n},\;$$

where $d_{1i}\in Dis_{1}$ and $d_{2j}\in Dis_{2}$.

### 2.4. Gaussian Interaction Profile (GIP) Kernel Simlarity

Previous studies [29,30,31] show that GIP kernel similarity can be constructed from known LDAs to increase the topology structure of the LDA network. The similarity score between disease $d_{i}$ and $d_{j}$ can be defined as following:

(6) $$K_{D}\left(d_{i},d_{j}\right)=\exp\left(-\gamma_{d}{∥Y\left(d_{i}\right)-Y\left(d_{j}\right)∥}^{2}\right).\;$$

      The lncRNA network similarity between $l_{i}$ and $l_{j}$ can be obtained in a similar way:

(7) $$K_{L}\left(l_{i},l_{j}\right)=\exp\left(-\gamma_{l}{∥Y\left(l_{i}\right)-Y\left(l_{j}\right)∥}^{2}\right),\;$$

where $\gamma_{d}$ and $\gamma_{l}$ are the parameters that control the kernel bandwidth. In this study, $\gamma_{d}=\frac{\sum_{i=1}^{\mu }{∥Y(d_{i})∥}^{2}}{\mu },$ and $\gamma_{l}=\frac{\sum_{i=1}^{\nu }{∥Y(l_{i})∥}^{2}}{\nu }.$$Y(d_{i})$ and $Y(d_{j})$ are the disease interaction profiles. $Y(d_{i})$ denotes the *i*th row vector in the incidence matrix. μ is number of diseases in the data set. $Y(l_{i})$ and $Y(l_{j})$ denote the lncRNA interaction profiles. $Y(l_{i})$ denotes the *i*th column vector in the incidence matrix. ν is number of diseases in the data set.

Relevant studies [29,32] have shown that logistic function transformation can improve the predictive ability of disease-associated problems. Therefore, we take the logistic function transform for $K_{D}$ and $K_{L}$:

(8) $$L_{D}\left(d_{i},d_{j}\right)=\frac{1}{1+e^{c\cdot K_{D}\left(d_{i},d_{j}\right)+x}},\;$$

(9) $$L_{L}\left(l_{i},l_{j}\right)=\frac{1}{1+e^{c\cdot K_{L}\left(l_{i},l_{j}\right)+x}}.\;$$

      The value of parameter *x* is set to log(9999) in line with the previous study [30]. The parameter *c* is tuned by the experiments.

### 2.5. Similarity Fusion

Disease semantic similarity and disease GIP kernel similarity are linearly fused to obtain the fused disease similarity matrix, and lncRNA functional similarity and lncRNA GIP kernel similarity are linearly fused to obtain the fused disease similarity matrix.

(10) $$F_{D}=f_{1}S_{dis}+f_{2}L_{D},\;$$

(11) $$F_{L}=f_{1}S_{l}+f_{2}L_{L}.\;$$

### 2.6. WKNKN Preprocessing

There may be some potentially unknown interactions in the known LDA matrix. In this study, the WKNKN method is used to initialize the association probabilities for potential interactions [33]. Specifically, the 0 values in the known LDA matrix are replaced by the values between 0 and 1 by the following steps:

(1) The *K* nearest neighbors are picked out by *K*-nearest neighbor (KNN) algorithm for each disease $d_{j}$, and they are arranged in a descending order. The weighted average of the similarities between the disease $d_{j}$ and its *K* nearest neighbors can be obtained as follows:

(12) $$Y_{d}\left(:,d_{j}\right)=\frac{1}{Z_{d}}\sum_{nd=1}^{K}w_{nd}Y_{d}\left(:,d_{nd}\right),\;$$

where $w_{nd}=\eta^{nd-1}F_{D}\left(d_{nd},d_{j}\right)$ denotes the weight coefficient, η⩽1 is a delay factor, and $Z_{d}=\sum_{nd=1}^{K}F_{D}\left(d_{nd},d_{j}\right)$ is the normalization term.

(2) Similarly, the weighted average of the similarities between the lncRNA $l_{i}$ and its *K* nearest neighbors can be calculated as follows:

(13) $$Y_{l}\left(l_{i},:\right)=\frac{1}{Z_{l}}\sum_{nl=1}^{K}w_{nl}Y_{l}\left(l_{nl},:\right),\;$$

where $w_{nl}=\eta^{nl-1}F_{L}\left(l_{i},l_{nl}\right)$ is the weight coefficient, η⩽1 is a delay factor, and $Z_{l}=\sum_{nl=1}^{K}F_{L}\left(l_{i},l_{nl}\right)$ is the normalization term.

(3) The zero entries in the known LDA matrix Y are replaced by the averages of $Y_{d}$ and $Y_{l}$. Then, $Y_{i,j}$ denotes the probability that the lncRNA $l_{i}$ is related to the disease $d_{j}$ and it can be defined as follows:

(14) $$Y_{i,j}=\left\{\begin{array}{cc} \frac{Y_{d}+Y_{l}}{2}, & if\;Y_{i,j}=0\\ Y_{i,j}, & if\;Y_{i,j}\neq 0 \end{array}\right..\;$$

### 2.7. Linear Neighborhood Similarity (LNS)

Roweis et al. [34] discovered that a data point and its neighboring data points are close to the locally linear patch of the manifold in a feature space. Wang et al. [35] revealed that each data point can be reestablished by its neighbors. In recent years, some researchers [18,36,37] obtained the pairwise similarity by reconstructing the data point through its neighbors. Here, we calculate the similarity between two different lncRNA data points (or two different disease data points) as previous work. Let $x_{i},i=1,\cdots ,nl$ denote the feature vector of the lncRNA $l_{i}$ in a feature space. Assume that the data point $x_{i}$ can be reestablished by the linear combination of its neighbors, we write the objective function and minimize the reconstruction error as follows:

(15) $$\begin{array}{cc} \varepsilon_{i} & ={∥x_{i}-\sum_{i_{j}:x_{i_{j}}\in N\left(x_{i}\right)}w_{i,i_{j}}x_{i_{j}}∥}^{2}+\lambda {∥w_{i}∥}^{2}\\ \; & =\sum_{i_{j},i_{k}:x_{i_{j}},x_{i_{k}}\in N\left(x_{i}\right)}w_{i,i_{j}}G_{i_{j},i_{k}}^{i}w_{i,i_{k}}+\lambda {∥w_{i}∥}^{2}\\ \; & =w_{i}^{T}G^{i}w_{i}+\lambda \sum_{x_{i_{j}\in N\left(x_{i}\right)}}{\left(w_{i,i_{j}}\right)}^{2}\\ \; & =w_{i}^{T}\left(G^{i}+\lambda I\right)w_{i} \end{array},\;$$

$$s.t.\sum_{i_{j}:x_{i_{j}}\in N\left(x_{i}\right)}w_{i,i_{j}}=1,w_{i,i_{j}}⩾0,j=1,\cdots ,K.$$

where $N(x_{i})$ is the set of  K(0<K<nl) nearest neighbors of the node $x_{i}$. $x_{i_{j}}$ is the *j*-th neighbor of $x_{i}$. $w_{i}={\left(w_{i,i_{1}},w_{i,i_{2}},\cdots ,w_{i,i_{K}}\right)}^{T}$, and $w_{i,i_{j}}$ is the reconstructive weight of $x_{i}$ from $x_{i_{j}}$. $G^{i}\in R^{K\times K}$ and $G_{i_{j},i_{k}}^{i}={\left(x_{i}-x_{i_{j}}\right)}^{T}\left(x_{i}-x_{i_{k}}\right)$. The regularization parameter λ is very important for the optimization problem (13). In this paper, the parameter λ is set to 1 based on the study of Ref. [37].

The optimization problem for each data point $x_{i}$ can be solved by using the standard quadratic programming technique. Finally, the weight matrix $W_{l}$ with size nl×nl can be obtained, which describes the pairwise similarity between nl lncRNAs. The weight matrix $W_{d}$ can also be calculated in the same way, which denotes the pairwise similarity between nd diseases.

### 2.8. Unbalanced Bi-Random Walk

Inspired by the successful applications of bi-random walks in identifying drug-disease associations [38], predicting miRNA-disease associations [39] and inferring LDAs [18], we design a novel method (called MSF-UBRW) based on unbalanced bi-random walks on the DSN and the LSN to identify potential LDAs. First, a bipartite G(V,E) is used to represent LDAs. *V* denotes the set of vertices, and *E* is the set of edges. The weight of edge $e_{ij}$ is equal to 1 when the disease $d_{i}$ is related to the lncRNA $l_{j}$, otherwise $e_{ij}=0$. Next, there are many isolated nodes in the DSN and the LSN. In this study, LNS is used to overcome this shortcoming. Finally, based on the assumption that similar diseases may be related to similar lncRNAs, and vice versa, unbalanced bi-random walks are executed on the DSN and the LSN simultaneously. Considering the differences in the topology of the two networks, different random walk steps are performed on the DSN and the LSN.

The column-normalized adjacency matrix $M_{D}\in R^{n_{d}\times n_{d}}$ of the DSN can be defined as:

(16) $$M_{D}\left(i,j\right)=\left\{\begin{array}{ccc} \frac{W_{d}\left(i,j\right)}{\sum_{p=1}^{n_{d}}W_{d}\left(p,j\right)}, & & \mathrm{if}\;\sum_{p=1}^{n_{d}}W_{d}\left(p,j\right)\neq \;0\\ 0, & & \mathrm{otherwise}. \end{array}\right.\;$$

The column-normalized adjacency matrix $M_{L}\in R^{n_{l}\times n_{l}}$ of the LSN can be calculated as:

(17) $$M_{L}\left(i,j\right)=\left\{\begin{array}{ccc} \frac{W_{l}\left(i,j\right)}{\sum_{p=1}^{n_{l}}W_{l}\left(p,j\right)}, & & \mathrm{if}\;\sum_{p=1}^{n_{l}}W_{l}\left(p,j\right)\neq \;0\\ 0, & & \mathrm{otherwise}. \end{array}\right.\;$$

Let $P\in R^{n_{d}\times n_{l}}$ denote the association probability matrix. The element P(i,j) is the probability that the disease *i* is associated with the lncRNA *j*. $s_{1}$ and $s_{2}$ denote the steps of random walks on the DSN and the LSN, respectively. The iterative process of bi-random walks can be defined as follows:

$$\mathrm{DSN}:\;D_{P}^{\left(t+1\right)}=\left(1-\alpha \right)\cdot P^{\left(t\right)}\cdot M_{D}+\alpha \cdot Y,$$

$$\mathrm{LSN}:\;L_{P}^{\left(t+1\right)}=\left(1-\alpha \right)\cdot M_{L}\cdot P^{\left(t\right)}+\alpha \cdot Y,$$

where α is a delay factor with a value ranging from 0.1 to 0.9. *t* denotes the number of iterations. ***Y*** denotes the known association information. $P^{\left(0\right)}$ is the initial association probability matrix, and $P^{\left(0\right)}=Y=Y/sum\left(Y\left(:\right)\right)$.

**Figure 2:**
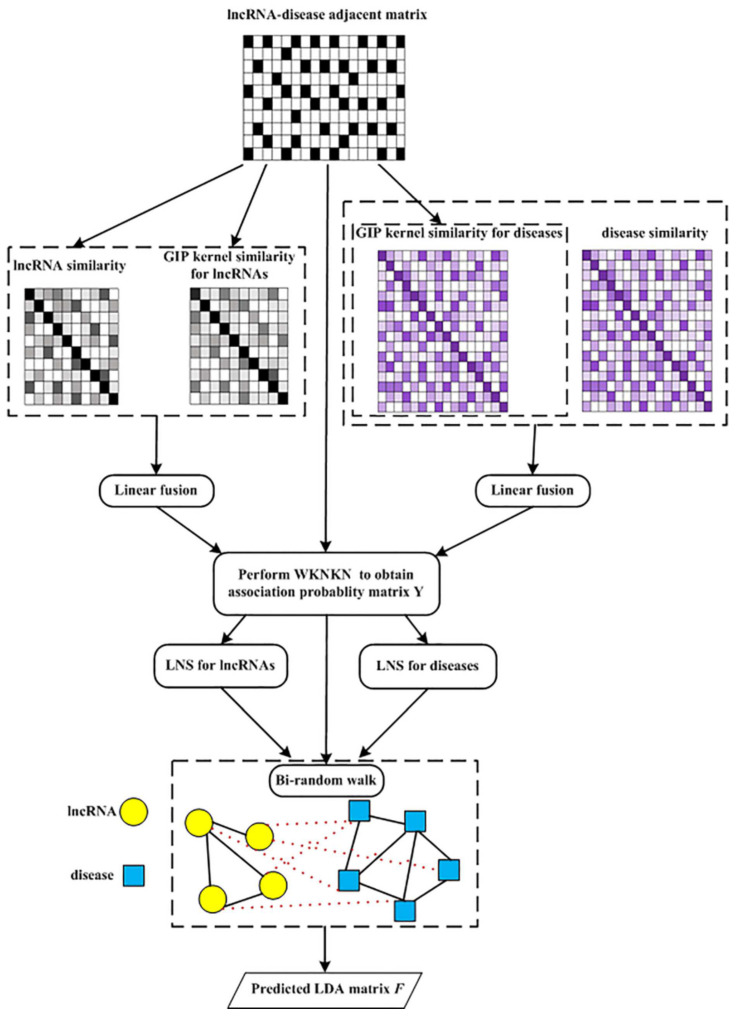
Flowchart of MSF-UBRW.

The flowchart of the MSF-UBRW algorithm is shown in Figure 2, and its pseudocode is Algorithm 1.
**Algorithm 1** MSF-UBRW**Input:** Known association information Y, parameters *K*, *c*, $s_{1}$, $s_{2}$, η and α**Output:** final LDA matrix F  1:GIP kernel similarity $K_{L}$ for lncRNAs;  2:GIP kernel similarity $K_{D}$ for diseases;  3:The logistic function $L_{L}$ for lncRNAs;  4:The logistic function $L_{D}$ for diseases;  5:Linear fusion: $F_{D}=f_{1}S_{dis}+f_{2}L_{D}$;  6:Linear fusion: $F_{L}=f_{1}S_{l}+f_{2}L_{L}$;  7:Pre-processing: $Y=WKNKN(Y,F_{D},F_{L},K,\eta )$;  8:The lncRNA similarity matrix $W_{l}$ based on LNS;  9:The disease similarity matrix $W_{d}$ based on LNS;10:Initialization: F=0;11:$P_{0}=Y/sum\left(Y\left(:\right)\right)$;12:Regularization:$M_{D}\left(i,j\right)=\frac{W_{d}\left(i,j\right)}{\sum_{p=1}^{n_{d}}W_{d}\left(p,j\right)}$, if  $\sum_{p=1}^{n_{d}}W_{d}\left(p,j\right)\neq 0.$Otherwise, $M_{D}\left(i,j\right)=0$.$M_{L}\left(i,j\right)=\frac{W_{l}\left(i,j\right)}{\sum_{p=1}^{n_{l}}W_{l}\left(p,j\right)}$, if $\sum_{p=1}^{n_{l}}W_{l}\left(p,j\right)\neq 0$.Otherwise, $M_{L}\left(i,j\right)=0$.13:$Iter=\max(\left[s_{1},s_{2}\right])$; //Iteration14:for p=1:Iter15:$r_{D}=0$;16:$r_{L}=0$;17://Bi-randomly walking;18:if $p<=s_{1}$19:$D_{P}^{\left(t+1\right)}=\left(1-\alpha \right)\cdot P^{\left(t\right)}\cdot M_{D}+\alpha \cdot Y$;20:$r_{D}=1$;21:end22:if $p<=s_{2}$23:$L_{P}^{\left(t+1\right)}=\left(1-\alpha \right)\cdot M_{L}\cdot P^{\left(t\right)}+\alpha \cdot Y$;24:$r_{L}=1$;25:end26:$P^{\left(t+1\right)}=\left(r_{D}\cdot D_{P}^{\left(t+1\right)}+r_{L}\cdot L_{P}^{\left(t+1\right)}\right)/\left(r_{D}+r_{L}\right)$;27:end28:$F=P^{\left(t+1\right)}$;29:Return F;

## 3. Results

### 3.1. Performance Evaluation

In order to evaluate the performance of the MSF-UBRW method in predicting undiscovered LDAs, 5-fold CV and LOOCV are performed on the gold standard dataset downloaded from the LncRNADisease database [28]. In 5-fold CV, all known LDAs are randomly divided into 5 parts. Each part serves as the testing samples in turn and the others as the training samples. In this experiment, 5-fold CV is run 100 times to take the average value. In LOOCV, each known LDA is treated as the test sample in turn, and the remaining known LDAs are treated as the training samples. In 5-fold CV and LOOCV, the test samples are compared with all unknown LDAs. Area Under Curve (AUC) is the final evaluation metric. Previous studies [21] have shown that this method is meaningless when AUC is between 0 and 0.5. When AUC lies between 0.5 and 1, the larger the AUC value is, the better the prediction performance of this method will be.

### 3.2. Comparison with Other Methods

**Figure 3:**
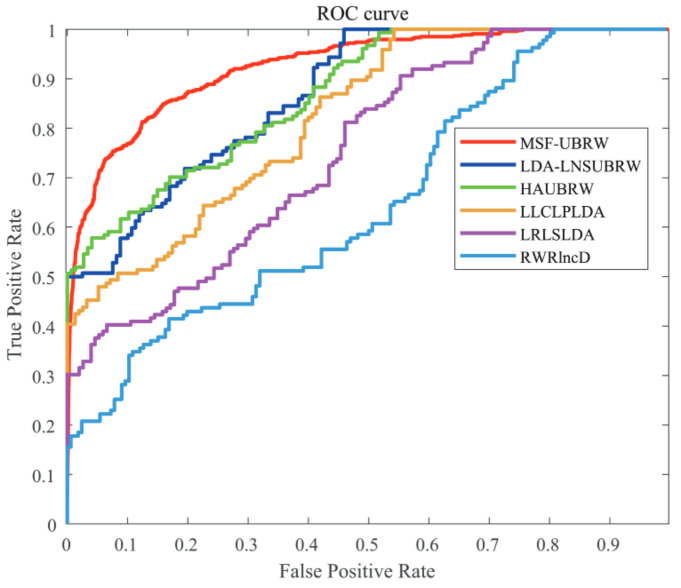
The ROC curves of the six methods (MSF-UBRW, LDA-LNSUBRW, HAUBRW, LLCLPLDA, LRLSLDA and RWRlncD) based on the 5-fold CV method.

**Figure 4:**
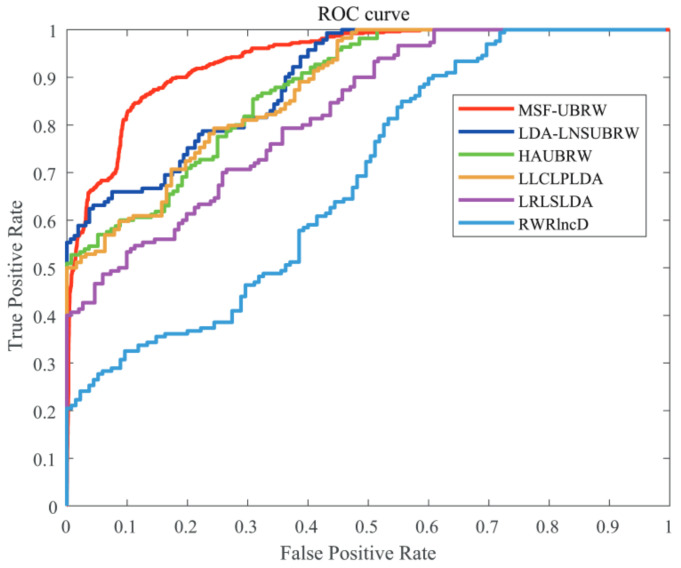
The ROC curves of the six methods (MSF-UBRW, LDA-LNSUBRW, HAUBRW, LLCLPLDA, LRLSLDA and RWRlncD) based on the LOOCV method.

In this paper, the MSF-UBRW method is compared with the other five prediction methods, namely, LDA-LNSUBRW [18], HAUBRW [27], LLCLPLDA [26], LRLSLDA [20], and RWRlncD [24]. First, the MSF-UBRW method is compared with these prediction methods in 5-fold CV. The AUC values of these six methods are shown in Table 1. The MSF-UBRW method achieves the AUC value of 0.9183(±0.0054), which is higher than the AUC values of the other methods (LDA-LNSUBRW: 0.8632(±0.0051), HAUBRW: 0.8617(±0.0064), LLCLPLDA: 0.8153(±0.0046), LRLSLDA: 0.7448(±0.0041) and RWRlncD: 0.6425(±0.0051)). Table 1 also presents the prediction results of the MSF-UBRW method and other five methods (LDA-LNSUBRW, HAUBRW, LLCLPLDA, LRLSLDA, and RWRlncD) via LOOCV. The MSF-UBRW method performs the best in predicting LDAs and its AUC value achieves 0.9391, which exceeds the other five methods (LDA-LNSUBRW: 0.8874, HAUBRW: 0.8693, LLCLPLDA: 0.8678, LRLSLDA: 0.8174 and RWRlncD: 0.6804). Figure 3 and Figure 4 show intuitively the comparison of the prediction performance of these six methods in 5-fold CV and LOOCV, respectively.

### 3.3. Parameters Analysis

**Figure 5:**
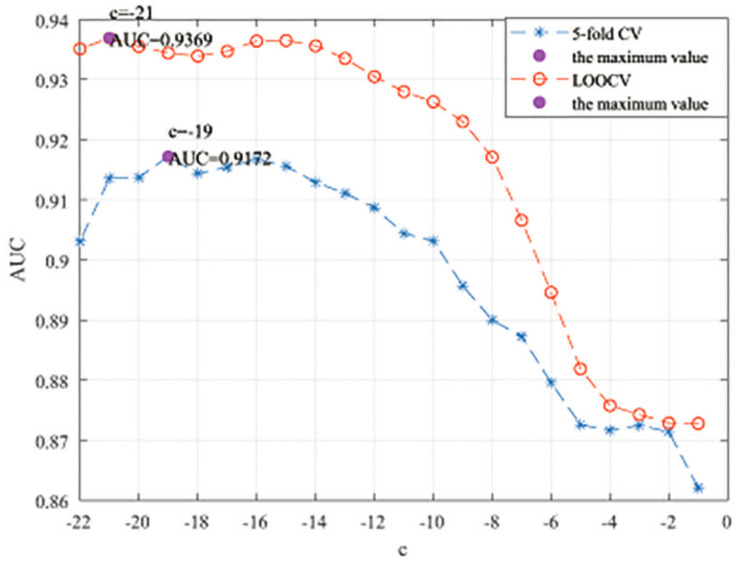
Sensitivity analysis of parameter *c*.

**Figure 6:**
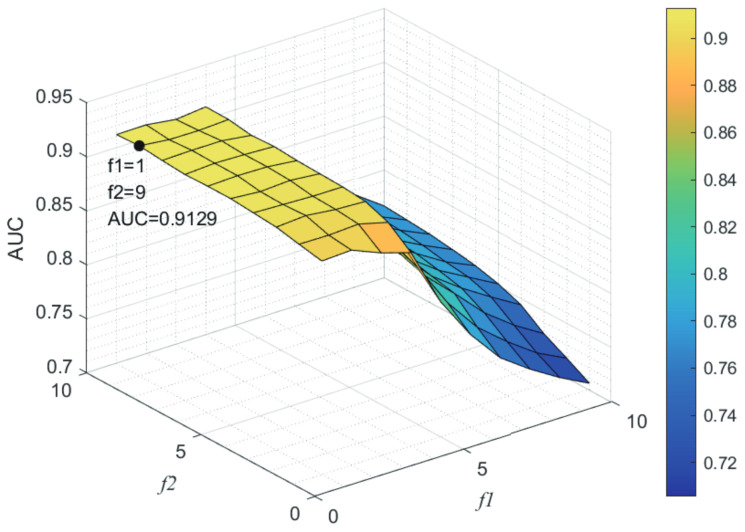
Sensitivity analysis of parameter $f_{1}$ and $f_{2}$.

**Figure 7:**
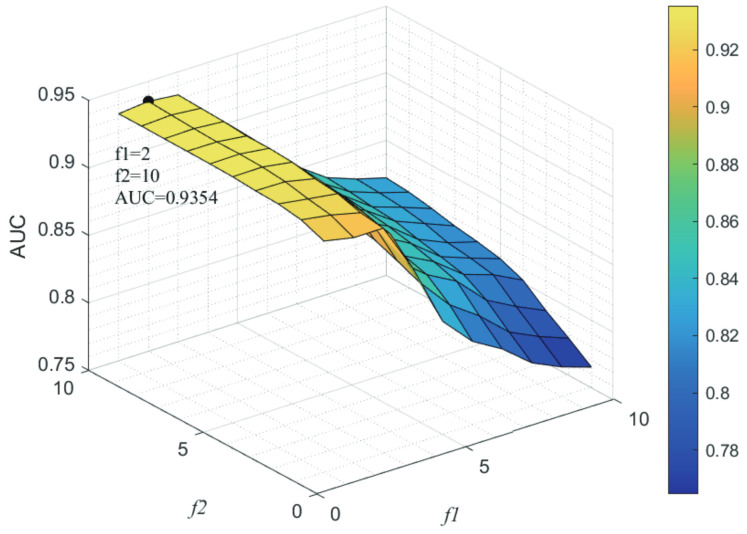
Sensitivity analysis of parameter $f_{1}$ and $f_{2}$.

**Figure 8:**
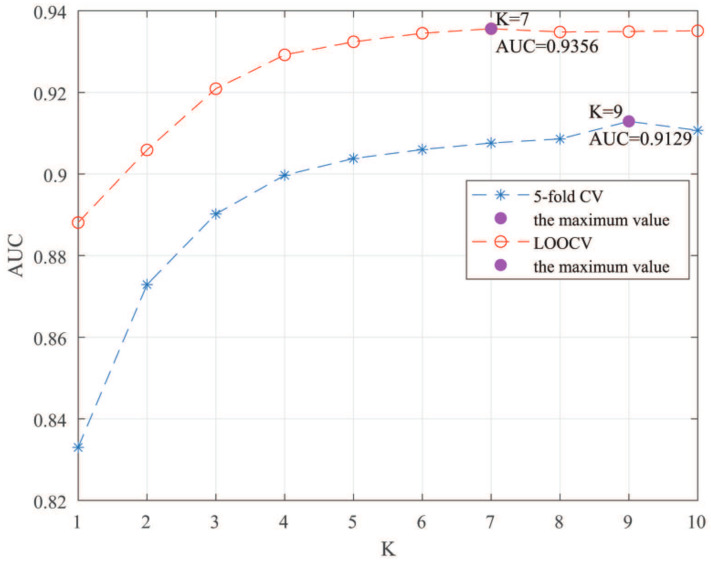
Sensitivity analysis of parameter *K*.

**Figure 9:**
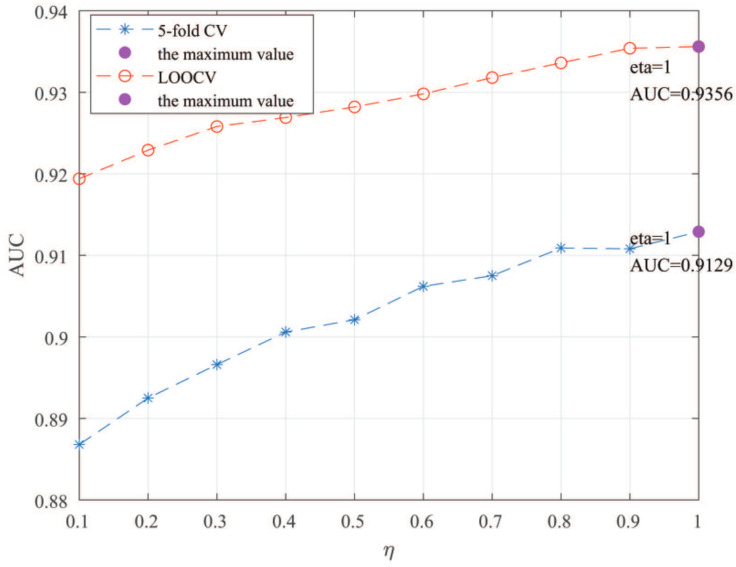
Sensitivity analysis of parameter η.

**Figure 10:**
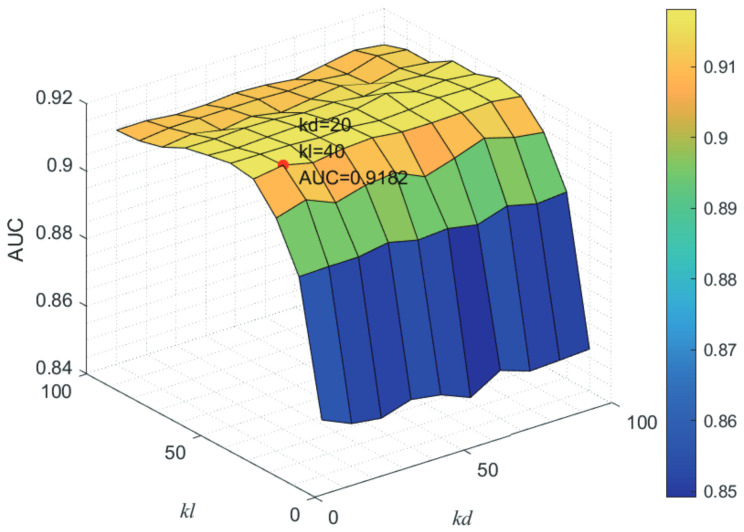
Joint sensitivity analysis of parameters $k_{l}$ and $k_{d}$.

**Figure 11:**
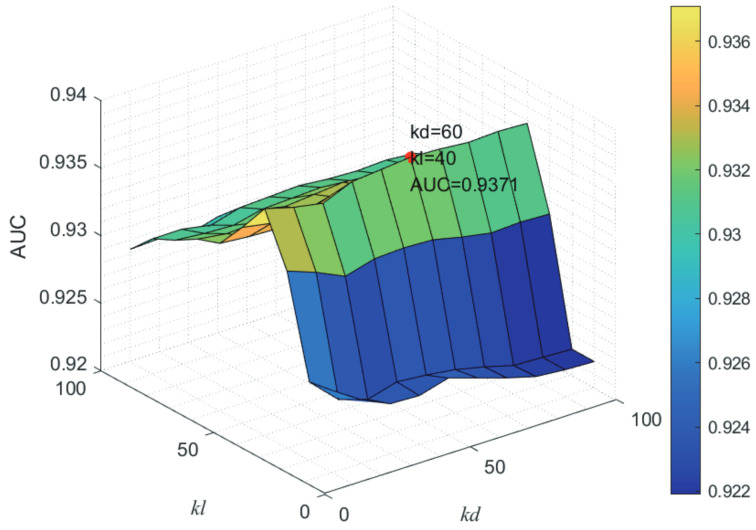
Joint sensitivity analysis of parameters $k_{l}$ and $k_{d}$.

**Figure 12:**
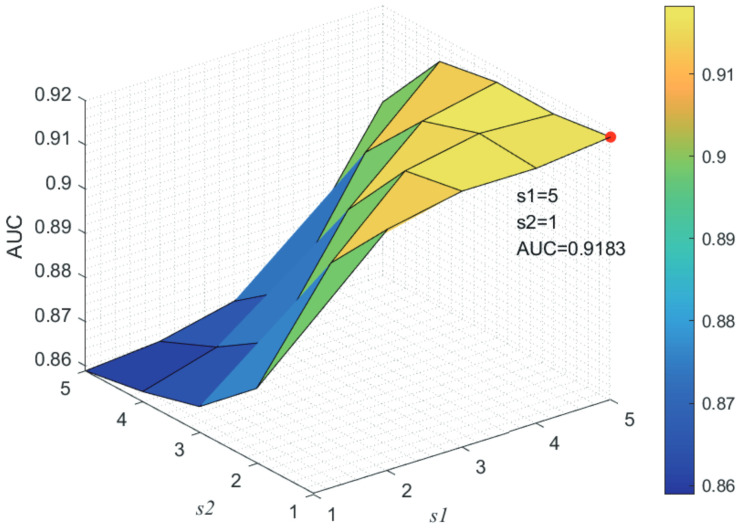
Joint sensitivity analysis of parameters $s_{1}$ and $s_{2}$.

**Figure 13:**
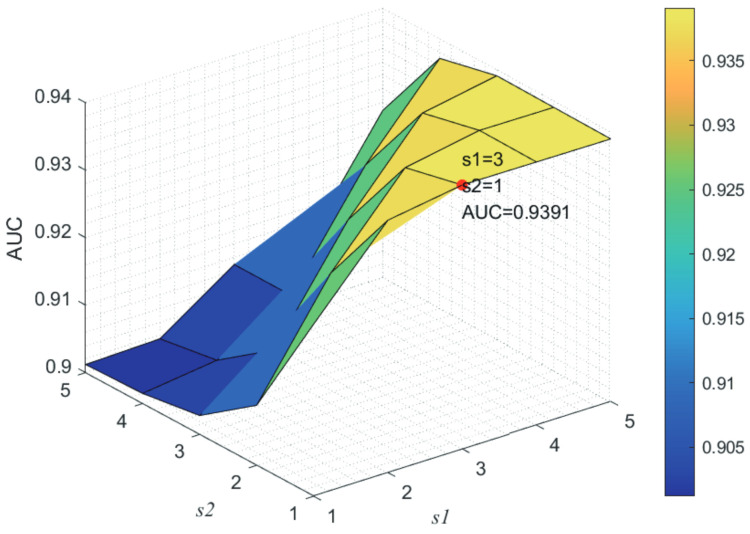
Joint sensitivity analysis of parameters $s_{1}$ and $s_{2}$.

**Figure 14:**
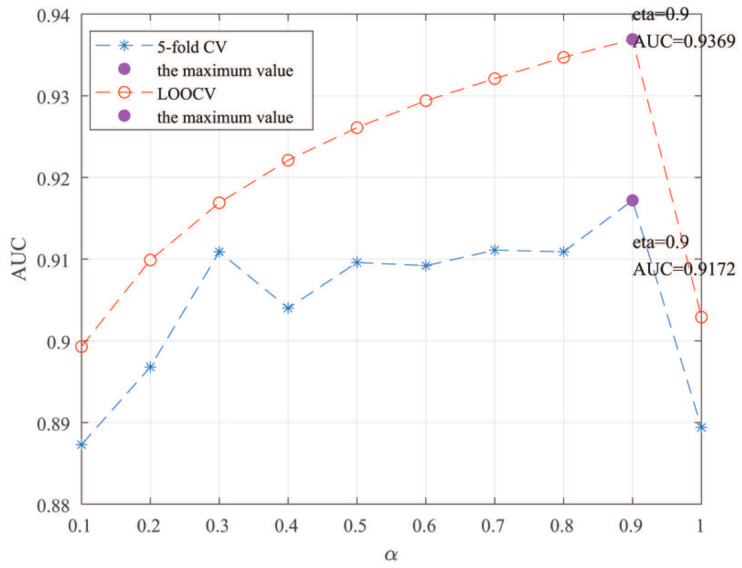
Sensitivity analysis of parameter α.

Here, we use the 5-fold CV and LOOCV to select the most appropriate parameters in the MSF-UBRW method. First, for the parameter *c* in the logistic function, it ranges from −1 to −21. From Figure 5, we can see that MSF-UBRW can gain the best prediction performance when *c* is equal to −19 in 5-fold CV and −21 in LOOCV. As shown from Figure 6, $f_{1}$ and $f_{2}$ is set to 1 and 9 in 5-fold CV, respectively. According to Figure 7, $f_{1}$ and $f_{2}$ is set to 2 and 10 in LOOCV, respectively. Next, for the number of known nearest neighbors *K* and the delay factor η in WKNKN, *K* is adjusted from 1 to 10 and η is adjusted from 0.1 to 1. According to Figure 8 and Figure 9, we finally set K=9 and η=1 in 5-fold CV, while K=7 and η=1 in LOOCV. Third, for the number of lncRNA neighbors $k_{l}$ and the number of disease neighbors $k_{d}$ in LNS, they are adjusted from 10 to 100, increasing by 10 each time. In fact, the number of lncRNA neighbors is less than the total number of lncRNAs, and the same is true for diseases. Considering the computational complexity, the maximum value of $k_{l}$ and $k_{d}$ is set to 100. As shown from Figure 10, $k_{l}$ and $k_{d}$ is set to 40 and 20 in 5-fold CV, respectively. According to Figure 11, $k_{l}$ and $k_{d}$ is set to 40 and 60 in LOOCV, respectively. Finally, we determine the maximum numbers of bi-random walks steps $s_{1}$ and $s_{2}$ on DSN and LSN. A grid searching method is conducted to analyze the parameters $s_{1}$ and $s_{2}$ via 5-fold CV and LOOCV. As seen from Figure 12 and Figure 13, the MSF-UBRW method achieves the highest AUC values when $s_{1}=5$ and $s_{2}=1$ in 5-fold CV and $s_{1}=3$ and $s_{2}=1$ in LOOCV. There is also a delay factor α in the bi-random walk algorithm. α is adjusted from 0.1 to 0.9. The prediction performance as α changes as shown in Figure 14. Obviously, α should be equal to 0.9 in both 5-fold CV and LOOCV.

### 3.4. Case Studies

To further verify the prediction ability of the MSF-UBRW method, case studies of human diseases are performed in this section. Three common cancers are selected for verification: prostate cancer, ESCC, and NSCLC. The final prediction matrix is obtained by the MSF-UBRW method. The predicted scores are ranked in descending order for the column and the top 20 lncRNAs are selected for analysis. The prediction results are validated by two databases: Disease v2.0 (http://www.rnanut.net/lncrnadisease/) and Lnc2Cancer 3.0/ (http://bio-bigdata.hrbmu.edu.cn/lnc2cancer/).

Prostate cancer is caused by malignant hyperplasia of prostate epithelial cells with a very high incidence of the urinary system. It is closely related to age. The older the age, the higher the incidence. The early symptoms of the disease are not obvious, and the symptoms of metastasis are prone to appear, which will endanger the life of the patients. The top 20 lncRNAs with higher predicted scores related to prostate cancer are listed in descending order in Table 2. From Table 2, we can find that 13 known LDAs in the gold standard dataset are predicted successfully. We use the database LncRNADisease v2.0 and Lnc2Cancer 3.0 to verify whether the other 7 lncRNAs are associated with prostate cancer.

Recent studies [40] revealed that the CDKN2B-AS1 is overexpressed in prostate cancer. Du et al. [41] found that XIST is down-regulated in prostate cancer specimens and cell lines, and has a tumor suppressor effect in prostate cancer. Its regulatory role will provide new ideas for epigenetic diagnosis and treatment of prostate cancer. Huo et al. [42] demonstrated that BCYRN1 was overexpressed in prostate tumors. Some studies [43,44] revealed PTENP1 may act to suppress prostate cancer. So far, NPTN-IT1 and BOK-AS1 have not been found to be related to prostate cancer.

ESCC belongs to the category of esophageal malignant tumors. The main symptoms of ESCC are pain and difficulty swallowing after eating hard and dry food, which brings great pain to the patients. The cause of ESCC is not yet fully understood, and its treatment remains a worldwide problem till now. From Table 3, we can see that 13 known LDAs are predicted successfully. By searching in the database LncRNADisease v2.0 and Lnc2Cancer 3.0, six lncRNAs (GAS5, MEG3, PVT1, NEAT1, XIST and CCAT1) associated with ESCC are confirmed. Wang et al. [45] found that the expression of GAS5 was significantly reduced in ESCC patients and it can act as a tumor suppressor factor. Huang et al. [46] revealed that MEG3 decreased significantly in ESCC tissues. Zhang et al. [47] reported that the lncRNA CCAT1 was significantly up-regulated in ESCC tissues compared with normal tissues, and it was related to the prognosis. The up-regulation of XIST expression promoted the proliferation of ESCC cells [48]. Besides, PVT1 and NEAT1 were also verified to be related to ESCC [49,50,51,52]. BCYRN1 has not been confirmed to be associated with ESCC.

Lung cancer is currently the cancer that causes the highest mortality among malignant tumors in China. Compared to small cell lung cancer, NSCLC develops and spreads more slowly, but it is usually found to be very advanced and difficult to control and treat. There are 15 lncRNAs associated with NSCLC in the oringinal dataset. In this experiment, all these 15 lncRNAs have been confirmed to be associated with NSCLC. LncRNAs H19, CDKN2B-AS1, BCYRN1, UCA1 and LSINCT5 are demonstrated to be associated with NSCLC in the database LncRNADisease v2.0 and Lnc2Cancer 3.0. Evidences that these four lncRNAs are related to NSCLC are shown in Table 4 [53,54,55,56,57,58,59,60]. There is no evidence to prove that CDKN2B-AS1 is associated with NSCLC.

## 4. Conclusions

More and more studies have found that changes in lncRNA expression patterns are associated with specific diseases. Building computational models to predict LDAs is not only a meaningful complement to experimental methods, but also helps researchers to gain insight into the pathogenesis of diseases. In this study, based on GIP and LNS, MSF-UBRW performs unbalanced bi-random walks in the LSN and DSN based on multiple similarities fusion to find new LDAs. Compared with LDA-LNSUBRW, HAUBRW, LLCLPLDA, LRLSLDA, and RWRlncD methods, the MSF-UBRW method achieves the highest AUC values under 5-fold CV and LOOCV. In addition, case studies of prostate cancer, ESCC, and NSCLC also confirm the prediction ability of the MSF-UBRW method.

Although the MSF-UBRW method has achieved good prediction results, it still have some limitations. Existing experimental data are inadequate, which limits the prediction performance of the MSF-UBRW method. In the future, as more LDA data are available, the MSF-UBRW method will be improved. However, the complexity and heterogeneity of biological data also bring some difficulties in improving the prediction ability of the algorithm. In the future, we will integrate data from different sources and improve the integrity and quality of experimental data to achieve higher prediction performance.

## Figures and Tables

**Table 1:** Auc results of six methods.

Methods	Five-Fold CV	LOOCV
MSF-UBRW	0.9183(±0.0054)	0.9391
LDA-LNSUBRW	0.8632(±0.0051)	0.8874
HAUBRW	0.8617(±0.0064)	0.8693
LLCLPLDA	0.8153(±0.0046)	0.8678
LRLSLDA	0.7448(±0.0041)	0.8174
RWRlncD	0.6425(±0.0051)	0.6804

**Table 2:** Top 20 identified lncRNAs for prostate cancer.

Rank	lncRNA	Evidence
1	HOTTIP	LncRNADisease v2.0
2	H19	LncRNADisease v2.0
3	MALAT1	LncRNADisease v2.0
4	GAS5	LncRNADisease v2.0
5	MEG3	LncRNADisease v2.0
6	HOTAIR	LncRNADisease v2.0
7	KCNQ1OT1	LncRNADisease v2.0
8	UCA1	LncRNADisease v2.0
9	PVT1	LncRNADisease v2.0
10	HULC	Lnc2Cancer 3.0
11	DANCR	LncRNADisease v2.0
12	NEAT1	LncRNADisease v2.0
13	PCA3	LncRNADisease v2.0
14	CDKN2B-AS1	PMID: 31438464
15	XIST	PMID: 16261845;29212233
16	BCYRN1	PMID: 32705287
17	NPTN-IT1	unconfirmed
18	BOK-AS1	unconfirmed
19	PTENP1	PMID: 25461816;20577206
20	PCAT1	PMID: 22664915

**Table 3:** Top 20 identified lncRNAs for esophageal squamous cell carcinoma.

Rank	lncRNA	Evidence
1	H19	PMID:31551175
2	MALAT1	LncRNADisease v2.0
3	HOTAIR	LncRNADisease v2.0
4	UCA1	PMID: 30002691
5	TUG1	PMID: 31742924
6	CDKN2B-AS1	PMID: 25239644
7	MINA	unconfirmed
8	SPRY4-IT1	PMID: 27250657
9	HNF1A-AS1	PMID: 25608466
10	SOX2-OT	PMID: 24105929
11	CCAT2	PMID: 25919911
12	TUSC7	PMID: 29530057
13	FOXCUT	unconfirmed
14	GAS5	PMID: 29170131; 31866421
15	MEG3	PMID: 28405686; 28539329
16	BCYRN1	unconfirmed
17	PVT1	PMID: 33848670;28404954
18	NEAT1	PMID: 29147064; 26609486
19	XIST	PMID: 33345719
20	CCAT1	PMID: 27956498

**Table 4:** Top 20 identified lncRNAs for non-small cell lung cancer.

Rank	lncRNA	Evidence
1	GAS5	LncRNADisease v2.0
2	PVT1	LncRNADisease v2.0
3	MALAT1	LncRNADisease v2.0
4	HOTAIR	LncRNADisease v2.0
5	XIST	LncRNADisease v2.0
6	MEG3	LncRNADisease v2.0
7	NEAT1	LncRNADisease v2.0
8	CCAT2	LncRNADisease v2.0
9	BANCR	LncRNADisease v2.0
10	CCAT1	LncRNADisease v2.0
11	TUG1	LncRNADisease v2.0
12	HIF1A-AS1	PMID: 26339353
13	ADAMTS9-AS2	unconfirmed
14	LINC00261	Lnc2Cancer 3.0
15	PANDAR	LncRNADisease v2.0
16	H19	PMID: 30214583; 31219199
17	CDKN2B-AS1	PMID: 31775885
18	UCA1	PMID:31938341; 31951852
19	BCYRN1	PMID: 25866480; 32016455
20	LSINCT5	PMID: 29883241

## Data Availability

The datasets used in this study can be derived from the e LncRNADisease website (http://www.cmbi.bjmu.edu.cn/lncrnadisease).

## Abbreviations

The following abbreviations are used in this manuscript:

LDAs	lncRNA-disease associations
MSF-UBRW	multiple similarities fusion based on unbanlanced bi-random walk
GIP	Gaussian Interaction Profile
LOOCV	leave-one-out cross-validation
NMF	non-negative matrix factorization
LSN	lncRNA similarity network
DSN	disease similarity network
WKNKN	weighted K-nearest known neighbors
ESCC	esophageal squamous cell carcinoma
NSCLC	small cell lung cancer
